# Employees’ Views and Ethical, Legal, and Social Implications Assessment of Voluntary Workplace Genomic Testing

**DOI:** 10.3389/fgene.2021.643304

**Published:** 2021-03-17

**Authors:** Kunal Sanghavi, W. Gregory Feero, Debra J. H. Mathews, Anya E. R. Prince, Lori Lyn Price, Edison T. Liu, Kyle B. Brothers, J. Scott Roberts, Charles Lee

**Affiliations:** ^1^The Jackson Laboratory for Genomic Medicine, Farmington, CT, United States; ^2^Maine-Dartmouth Family Medicine Residency, Augusta, ME, United States; ^3^Berman Institute of Bioethics, Department of Genetic Medicine, Johns Hopkins University, Baltimore, MD, United States; ^4^College of Law, University of Iowa College of Law, Iowa City, IA, United States; ^5^The Institute for Clinical Research and Health Policy Studies, Tufts Medical Center, Boston, MA, United States; ^6^Tufts Clinical and Translational Science Institute, Tufts University, Boston, MA, United States; ^7^Department of Pediatrics, University of Louisville School of Medicine, Louisville, KY, United States; ^8^Department of Health Behavior and Health Education, School of Public Health, University of Michigan, Ann Arbor, MI, United States; ^9^Center for Bioethics and Social Sciences in Medicine, University of Michigan, Ann Arbor, MI, United States; ^10^Precision Medicine Center, The First Affiliated Hospital of Xi’an Jiaotong University, Xi’an, Shaanxi, China

**Keywords:** workplace genomic testing, workplace wellness, employees, ELSI, genetic health professionals

## Abstract

Employers have begun to offer voluntary workplace genomic testing (wGT) as part of employee wellness benefit programs, but few empirical studies have examined the ethical, legal, and social implications (ELSI) of wGT. To better understand employee perspectives on wGT, employees were surveyed at a large biomedical research institution. Survey respondents were presented with three hypothetical scenarios for accessing health-related genomic testing: via (1) their doctor; (2) their workplace; and 3) a commercial direct-to-consumer (DTC) genetic testing company. Overall, 594 employees (28%) responded to the survey. Respondents indicated a preference for genomic testing in the workplace setting (70%; 95% CI 66–74%), followed by doctor’s office (54%; 95% CI 50–58%), and DTC testing (20%; 95% CI 17–24%). Prior to participating in wGT, respondents wanted to know about confidentiality of test results (79%), existence of relevant laws and policies (70%), and privacy protection (64%). Across scenarios, 92% of respondents preferred to view the test results with a genetic counselor. These preliminary results suggest that many employees are interested and even prefer genetic testing in the workplace and would prefer testing with support from genetic health professionals. Confirmation in more diverse employer settings will be needed to generalize such findings.

## Introduction

Voluntary workplace genomic testing (wGT) in the context of wellness programs is a growing access point for genetic screening for disease risk (Singer, 2018). A 2017 Wamberg Genomic Consumer Survey of 536 U.S. consumers with employer-sponsored health insurance reported that 65% of employees would be interested in genetic testing if their employers offered easy and affordable access with strict privacy and data ownership, allowing test result access only to the employee and their doctor^1^. wGT has the potential to lower overall healthcare costs and improve health outcomes by encouraging employees to become more vigilant about disease risks, make lifestyle changes to manage these risks, and seek care before symptoms develop. Thus, wGT as part of wellness programs might be able to inform strategies to mitigate disease risks. However, whether wGT truly influences behavior change and offers long-term benefits remains an open question (Hollands et al., 2016; Burke et al., 2019; Yanes et al., 2019). Additionally, existing studies on wellness programs that report minimal benefits do not account for wGT, which is an emerging area (Lieberman, 2019; Song and Baicker, 2019).

Genetic screening in the workplace and the potential implementation of wGT as part of routine preventive medicine was envisioned as early as the 1980s (Dabney, 1981). Although wGT can inform employees of their genetic risk for certain treatable diseases, it also raises concerns about genetic privacy and workplace discrimination. There is also a recognition that the acquisition and use of genetic data in workplace settings might cause emotional distress and other psychosocial harms (MacDonald and Williams-Jones, 2002). Furthermore, the complex web of state and federal laws that regulate medical information privacy and employer-provided group health insurance coverage do not comprehensively ensure protections against inappropriate use or misuse of wGT by third parties, such as law enforcement or insurance companies (Annas, 2001; Drabiak, 2017; Hudson and Pollitz, 2017; Golinghorst and Prince, 2020). Such uncertainty and legal concerns add to the more direct concerns about how personal genomic data might be used in the workplace setting (Lappé, 1983; Van Damme and Casteleyn, 1998).

A better understanding of current factors affecting employee decision-making regarding participation in wGT will help inform research activities, education initiatives and policy development. This study reports on a survey of employees of a large, U.S. based, self-insured biomedical research institution with over 2,000 employees. Respondents were presented with hypothetical scenarios for accessing health-related genomic testing for screening purposes, then asked questions designed to: (a) ascertain interest in wGT compared to other access points for genetic testing, (b) assess employees’ perceived test concerns and benefits, and (c) determine sociodemographic, psychological, and situational correlates of test intentions.

## Materials and Methods

### Study Population

Participants were drawn from a convenience sample of employees of The Jackson Laboratory (JAX), a large biomedical research institution with 2,090 employees headquartered in Bar Harbor, ME with additional facilities in Sacramento, California, and Farmington, Connecticut. The study was voluntary and anonymous. The informed consent specified that study participation would have no negative consequences to respondents’ employment at JAX. Participants were not offered any inducement or incentive to participate in the study. The final version of the survey was reviewed by institutional regulatory and information security teams to ensure that study participation would not be perceived by employees as offering actual genomic testing and that employees’ privacy would be protected. The study was approved by JAX Institutional Review Board (IRB).

### Survey Development and Administration

An interdisciplinary team with expertise in legal and ethical issues of genetic testing, psychosocial outcomes, and clinical genetics developed a survey that included: (1) eighteen closed-ended questions; (2) one open-ended question; and (3) six demographic questions. Pilot testing with 8 JAX employees and health professionals helped improve clarity of survey items. Three hypothetical scenarios for accessing voluntary health-related genomic testing were presented, including testing through: (1) the participant’s doctor’s office; (2) an unspecified workplace (wGT); and (3) the participant’s engagement in direct-to-consumer (DTC) genetic testing. The scenarios included descriptions of variable factors related to genomic testing including cost, insurance implications, and sharing of test results. These variable factors were included to most closely reflect the current state of genetic screening across the three scenarios. Notably, genetic test results are most likely to go into medical records when testing occurs at a doctor’s office and direct-to-consumer testing is most likely to incur out of pocket expenses for an individual. Also, the hypothetical scenarios specifically mentioned the types of conditions that would be tested and did not include nutrition, fitness, or ancestry with an intention to promote health risk reduction relevant to all participants including those not taking any medications. A description of the testing was provided in the survey prior to the questions (see Supplementary Table S5). The complete survey is available in the online supplement (see Supplementary Table S1).

The survey was administered online following an email invitation in August 2018 (August 7–30) and was open to all JAX employees. Beyond the initial email, recruitment efforts included the following: (1) 1 week before the survey administration, paper handouts were displayed at designated sites on all three campuses along with a rolling display on wall-mounted video monitors in high-traffic common spaces throughout each campus; (2) a detailed description of the purpose of the study, types of questions, safeguards to protect employees’ privacy, contact information of the study team, and a set of Frequently Asked Questions were posted on the JAX internal website; and (3) reminder posts added to this website one and 3 weeks after emailing the survey link. These reminder posts were again supplemented by rolling announcements on video monitors in high-traffic common spaces on each JAX campus. The survey responses were compiled using QualtricsXM (August 2018, Provo, Utah, United States).

### Survey Measures

Sociodemographic characteristics collected by the survey included self-reported sex, age, years employed at JAX, highest education level completed, ethnicity, and race based on U.S. Census categories and examples from the National Institutes of Health (NIH). Additional information was collected regarding prior experience or interest in genomic testing; personal or family member history with genomic testing; the primary reason for seeking testing; and overall interest in genomic testing. Participants selected their level of agreement with a list of perceived benefits, risks, and limitations of genomic testing in the context of the three different scenarios using a 5-point Likert scale (1 = Strongly Disagree to 5 = Strongly Agree) (Table 3). Participants also had a choice to select “unsure” for each of the perceived benefits or concerns; for analyses these responses were combined with the neither agree/disagree response category. Prior research (Lerman et al., 1996) on predictive testing for other disorders was referenced to determine the risks and benefits selections available to participants.

Specific to the wGT scenario, participants were asked about questions that they would like answered prior to testing. Examples of such questions included those about: (a) impact on health, privacy and confidentiality; and (b) an opportunity to discuss wGT with a genetic counselor. Preferences for receiving results and factors influencing comfort or confidence with wGT were also assessed. A single open-ended question was included to elicit any additional thoughts on wGT that were not covered in the survey.

### Data Analysis

Descriptive statistics characterized participants in terms of their sociodemographics, predictive testing intentions, and concerns and perceived test benefits and concerns. Generalizability of the responses to the general employee population at JAX was assessed based on the available aggregate demographic information of the employees at JAX. A perceived test benefit score was derived by summing responses to four factors for pursuing testing. Similarly, a perceived test concern score was derived by summing responses to four factors for declining testing (see Table 3). Cronbach’s alpha test was used to evaluate the internal consistency of these summed scales. Logistic regression was used to determine whether there were associations between the test scenario and a positive response (probably or definitely yes) to whether the respondent would engage in genomic testing. Logistic regression was also used to test for associations between respondent characteristics and positive response (probably or definitely yes) to engaging in genetic testing in the three scenarios. Odds ratios (OR) and 95% confidence intervals (CI) are presented. *P*-values < 0.05 were considered statistically significant. Statistical analyses were conducted using SAS 9.4 (SAS Institute Inc., North Carolina, United States).

Free text responses to the question on factors influencing decision making for wGT were grouped into themes that were selected inductively based on an initial review of responses and refined through review by the broader study team. Themes were then applied to individual responses in an Excel spreadsheet. This approach was felt to be reasonable given that responses were generally brief and focused.

## Results

### Respondent Characteristics

Twenty eight percent (594/2,090) of all JAX employees (at the time of survey administration) consented to participate in the study of which 61% were female, 63% were older than 35, and 88% were White. Four percent (*N* = 20) of respondents preferred not to reveal their gender. Table 1 summarizes the respondents’ sociodemographic characteristics: sex, age, years employed at JAX, level of education completed, race, and ethnicity. In general, the demographic characteristics of respondents were similar to the overall employee population at JAX in age, sex, and race.

**TABLE 1:** Demographics of the respondents.

Sociodemographic characteristics	All respondents *N* (%) *N* = 594
**Sex (*N* = 496)^*a*^**	
Female	290(60.9)
Male	186(39.1)
**Age: (*N* = 489)**	
18–25	41(8.4)
26–35	140(28.6)
36–45	124(25.4)
46–55	119(24.3)
56 and above	65(13.3)
**Years at JAX: (*N* = 493)**	
Less than a year	75(15.2)
1–2	100(20.3)
3–5	116(23.5)
6–9	52(10.6)
10–19	99(20.1)
More than 20 years	51(10.3)
**Education: (*N* = 494)**	
High School or GED	25(5.1)
Some college/associate degree	109(22.1)
Bachelor’s degree	176(35.6)
Master’s degree	87(17.6)
Professional school/doctoral degree	97(19.6)
**Race: (*N* = 455)^*a*^**	
American Indian or Alaskan Native	5(1.1)
Asian	25(5.5)
Black or African American	4(0.9)
Native Hawaiian or other Pacific Islander	1(0.2)
White	399(87.7)
More than one race	21(4.6)
**Ethnicity: (*N* = 464)^*a*^**	
Hispanic	27(5.8)
Non-hispanic	437(94.2)

*^*a*^Prefer not to answer excluded and counted as missing.*

### Prior Experiences and Interest in Genomic Testing

Twenty two percent (130/593) of respondents reported that they had taken a genomic test in the past. Ancestry testing (42%) was the primary testing experience, followed by carrier testing (16%), and predictive testing (11%), and prenatal testing (8%). Twenty-one percent of respondents reported that their family members had taken a genomic test in the past, while 13% were unsure. Respondents indicated that their family member(s) had sought ancestry testing (52%), health-related diagnostic testing (13%), predictive testing (10%), or prenatal testing (10%).

### Perceived Benefits, Limitations, and Risks of Genetic Testing

Respondents were more likely to endorse test benefits than risks or concerns for each scenario (Table 2). Early diagnosis/detection had the highest number of respondents who agreed or strongly agreed that it was a benefit (≥85%) across the 3 test scenarios. Privacy/confidentiality had the highest number of respondents who agreed or strongly agreed that it was a concern (≥70%). Respondents were unsure whether potential work absenteeism due to follow-up care was a concern in wGT (4%), doctor’s office (4%), and DTC (6%) respectively. Regarding the perceived benefit vs. risk or concern of wGT, 80% of respondents agreed or strongly agreed with at least one listed perceived benefit to wGT while 71% agreed or strongly agreed with at least one concern raised by wGT.

**TABLE 2:** Perceived test benefits, risks and concerns.

	Test scenario			
	
	Employer based (% agree)	Doctor’s office (% agree)	Commercially available (% agree)	*p-*value
**Perceived benefit**				
Early diagnosis/detection	459/511 (90%)	485/529 (92%)	427/503 (85%)	<0.0001
Timely medical intervention	432/509 (85%)	460/529 (87%)	398/502 (79%)	<0.0001
Motivation for lifestyle change	440/510 (86%)	455/529 (86%)	402/501 (80%)	0.0001
Health risk info for children	411/511 (80%)	436/529 (82%)	370/502 (74%)	<0.0001
**Perceived risk/concern**				
Privacy/confidentiality	360/510 (71%)	421/528 (80%)	392/504 (78%)	<0.0001
Date use by outside group	331/509 (65%)	390/527 (74%)	384/503 (76%)	<0.0001
Misunderstanding of test	264/510 (52%)	285/529 (54%)	270/504 (54%)	0.39
Work absence	179/510 (35%)	172/528 (33%)	158/503 (31%)	0.10

*The frequencies and percentages in each row are based on those who responded “Agree” or “Strongly Agree” to the question using the Likert scale variables (5 categories strongly disagree to strongly agree). The numerators represent the total number of responses for “Agree” or “Strongly Agree” and denominators represent the total number of responses for each item in all 5 categories (strongly disagree to strongly agree). Generalized estimating equations models were used to test for associations of perceived benefits and risks across the three scenarios while accounting for the clustering within survey respondents.*

**TABLE 3:** Test interest by scenario.

	Scenario response: *Would you take this genomic screening test?*			
	
Test scenario	Not likely^*a*^ *N* (%)	Likely^*b*^ *N* (%)	OR (95% CI)	*p*-value
Employer-based (wGT^*c*^) (*N* = 512)	153 (29.9)	359 (70.1)	1.99 (1.63, 2.43)	<0.0001
Doctor’s office (*N* = 529)	243 (45.9)	286 (54.1)	Reference	
Commercially available (*N* = 506)	403 (79.6)	103 (20.4)	0.22 (0.17, 0.28)	<0.0001

*^*a*^Responses: Definitely not, Probably not, Might or might not.*

*^*b*^Responses: Probably yes, Definitely yes.*

*^*c*^wGT: voluntary workplace genomic testing.*

Thirty four percent of respondents preferred to learn about privacy and confidentiality safeguards followed by relevant laws and policies (∼30%) prior to taking the test. Perceptions of test benefits and risks are summarized in Table 3. Cronbach’s alpha was 0.91, 0.90, and 0.92 for wGT, doctor’s office, and DTC scenarios respectively for the benefits subscale. For the risks or concerns subscale, Cronbach’s alpha was 0.77, 0.63, and 0.72 respectively. This signifies the overall reliability of the scale items on perceived test benefits and risks or concerns. The percentage of respondents indicating agreement with the perceived benefit was lowest for the commercially available testing scenario for each of the four perceived benefits (*p* ≤ 0.0001) (see Table 2). However, the percent of agreement was high for three scenarios, indicating an overall endorsement of perceived benefits across all three scenarios. Fewer respondents were concerned about privacy and confidentiality concerns and data use by an outside group for the employer-based scenario (*p* < 0.0001). There were no significant differences across groups for perceived concerns of misunderstanding of test or work absence.

### Respondents’ Attitudes Toward wGT

Respondents were likely or highly likely to opt for genomic testing most commonly in the workplace setting (70%, 359/512, 95% CI 66–74%), followed by doctor’s office (54%, 286/529, 95% CI 50–58%), and DTC testing (20%, 103/506, 95% CI 17–24%). About 43% of respondents reported being most comfortable with the workplace among the three scenarios for genomic testing, followed by doctor’s office (25%), and DTC setting (9%). About 10% of respondents were uncomfortable with all three scenarios, while 9% were comfortable with all three scenarios. About 4% of respondents were unsure about their comfort with any of the three scenarios. Respondents had increased odds of being likely (probably or definitely) to take wGT (OR 1.99, 95% CI 1.63–2.43, *p* < 0.0001) and reduced odds to use commercially available tests (OR 0.22, 95% CI 0.17–0.28, *p* < 0.0001) compared to taking the test in the doctor’s office. A summary of test preferences in the three hypothetical scenarios appears in Table 3. In our cohort, respondents with an associate degree or less had an odds ratio of 3.37 for interest in wGT as compared to those with a Doctor of Philosophy (Ph.D) or Doctor of Medicine (MD). This trend was seen in intermediate levels of education: Bachelor’s degree (OR 2.85) and Master’s degree (OR 1.15).

### Factors Affecting Preference for wGT

Twenty percent of respondents indicated that there were other factors that would make them more comfortable or confident about taking wGT, while 15% of respondents indicated that there might be some such factors. Across the free text responses (159 comments), the most common broad categories of factors were: (1) data access, ownership, and security, privacy and confidentiality; (2) effect on health insurance; (3) information on laws protecting privacy; and (4) job protection (see Table 4).

**TABLE 4:** Supporting respondents’ quotes for broad categories of factors impacting decision-making for wGT.

Broad categories of factors	Respondents’ quotes
Data access, ownership, and security, privacy and confidentiality	*“Assurance that the results would only be available to myself and a genetic counselor if I chose to disclose them.” “Genetic testing company would not be able to see information about me. Only the information I choose to put in my medical record would go, not my whole result file.” “Iron-clad guarantee that employer cannot recover individual info.” “Allow me to determine who has access to the information.”*
Effect on health insurance	*“Assurance that results do not increase healthcare cost or coverage and remain confidential.”*
Information on laws protecting privacy	*“Written details on privacy of information and laws protecting this information.”*
Job protection	*“Assurance of privacy and job protection.”*

*These broad categories of factors that might impact the decision-making for wGT were selected inductively using individual respondent responses. wGT, voluntary workplace genomic testing.*

Logistic univariate regression models assessing associations between demographics and positive response to taking wGT are described in Table 5. Younger age (*p* = 0.005) and lower education levels (*p* < 0.0001) were the only demographic variables with a statistically significant association with an expressed preference for wGT. Higher perceived benefits and lower perceived risks of wGT (except for understanding of test and work absence) were also associated with interest in wGT (see Supplementary Table S2). Additionally, in the genomic testing at doctor’s office scenario, a univariate logistic regression model revealed statistically significant associations with test interest for younger age (*p* < 0.0001), recent employment (<10 years) at JAX (*p* = 0.01), and lower education levels (*p* = 0.0006) (see Supplementary Table S3). Similar analysis for the DTC testing scenario did not reveal any significant associations (see Supplementary Table S4). Higher perceived benefits and lower perceived risks of doctor’s office-based testing (except for understanding of test and work absence) were also associated with interest in testing in a health care setting. Respondents who expressed lower perceived risks for understanding and work absence were less likely to express interest in taking the test at the doctor’s office. Higher perceived benefits and lower perceived risks of doctor’s office-based testing (except for understanding of test and work absence) were also associated with interest in the DTC testing scenario. Higher perceived benefits of early diagnosis and timely medical intervention and lower perceived risk of data used by outside group were associated with interest in the DTC testing scenario.

**TABLE 5:** Logistic univariate regression assessing predictors of interest in wGT.

Sociodemographic characteristics	OR (95% CI)
**Sex: (*N* = 475)^*a*^**	*P* = 0.49
Female	Reference
Male	1.16 (0.77, 1.76)
**Age: (*N* = 488)**	*P* = 0.005
18–35	2.43 (1.31, 4.51)
36–55	1.29 (0.73, 2.27)
56 and above	Reference
**Years at JAX: (*N* = 492)**	*P* = 0.20
Less than a year	1.83 (0.81, 4.15)
1–2	1.51 (0.71, 3.21)
3–5	0.94 (0.46, 1.90)
6–9	1.13 (0.49, 2.62)
10–19	0.84 (0.41, 1.72)
More than 20 years	Reference
**Education: (*N* = 493)**	*P* < 0.0001
Associate degree or less	3.37 (1.87, 6.06)
Bachelor’s degree	2.85 (1.67, 4.88)
Master’s degree	1.15 (0.64, 2.07)
Ph.D./MD	Reference
**Education: (*N* = 493)**	*P* < 0.0001
High school or GED	9.33 (2.08, 41.81)
Some college/associate degree	2.87 (1.57, 5.27)
Bachelor’s degree	2.85 (1.67, 4.88)
Master’s degree	1.16 (0.64, 2.07)
Professional school/doctoral degree	Reference
**Race: (*N* = 454)^*a*^**	*P* = 0.89
White	0.96 (0.51, 1.80)
Not white	Reference

*^*a*^Prefer not to answer excluded and counted as missing. Responses of unsure were included in the Strongly Disagree/Disagree/Neither agree nor disagree category for the analyses of perceived benefit and perceived risks.*

### Preferences Related to the Process of wGT

Of the three access points for genomic testing, 42% of respondents felt most comfortable with wGT. Regarding information that respondents wanted to know prior to participating in wGT, 79% selected information about confidentiality of test results, 70% of respondents selected existence of relevant laws and policies, and 64% of respondents selected privacy protection. Other topics that respondents expressed an interest in knowing more about included potential implications of the test result (50%) and possible impact of testing on their family (34%). Note that respondents could check multiple topics.

Of interest, ninety two percent of respondents agreed or strongly agreed that they preferred to view the test results with a genetic counselor, but only 38% of respondents preferred to discuss medical and family history with a genetic counselor before pursuing wGT. Ninety-nine percent of respondents agreed or strongly agreed they would like to receive a copy of their wGT results. Eighty-nine percent of respondents agreed or strongly agreed they would like to get a letter from a genetic counselor to learn the impact of their wGT test result on their health and potentially, their family’s health. With regard to preferences for how to receive a wGT test result, 58% of respondents ranked an in-person session with a genetic counselor as their top choice. A telephone session with a genetic counselor was second most popular choice (24%). A web-based session with a genetic counselor (28%) and an email link to a secure web portal with test results (25%) were the next most popular choices. A written summary of wGT test results was the least popular option, with more than a third (36%) selecting it as their fifth choice.

## Discussion

This preliminary study focused on developing a better understanding of employee attitudes regarding voluntary workplace genetic testing (wGT). Respondents, all of whom were employees of a large biomedical research institution focused on genetic research, expressed a significant preference for wGT over genetic testing for disease risk in the setting of a doctor’s office or through engagement of a DTC genetic testing company. As genetic testing gains public acceptance, and as its perceived utility for preventive care increases, questions arise as to the best venue for offering testing. This preference for workplace testing is significant and suggests that despite continued privacy concerns, non-clinical settings for genetic testing have likely gained wider acceptance than before. This is an important shift since, approximately 60% of non-elderly adults in the U.S. have medical coverage through employer-sponsored health insurance^2^. Of this group, 61% are covered by self-funded plans^3^, meaning that the employer pays the cost of healthcare for those covered. Preventive health programs, which may increasingly include wGT, offered through employer-sponsored plans are often incorporated into employer wellness programs. The confluence of financing, availability of genetic testing, and workforce acceptance may result in future sustainable growth of wGT.

The current wGT market is not entirely focused on medically actionable genetic testing as was described in the imaginary scenarios in the survey. This fact limits the generalizability of the findings to a particular subset of genomic testing that is currently available to many employees. However, the authors chose to focus on medically actionable findings in this study because such results are likely to be of particular interest to consumers, and are likely to raise concerns regarding discrimination if the results are improperly used.

One potential concern regarding widespread implementation of wGT is that many individuals may fear that their data could be used to discriminate against them. For example, many individuals fear discrimination by employers and insurers, even if they are aware of the provisions of the federal Genetic Information Non-discrimination Act (GINA) of 2008 that protects individuals from discrimination based on genetic information for employment and health insurance purposes (Green et al., 2015). Other concerns such as secondary use of the aggregated wGT results, might also affect implementation of wGT in wellness programs. For example, the unwanted or inappropriate sharing of aggregated wGT results could result in unintended consequences in the absence of sufficient privacy and confidentiality protections. The informed consent for wGT might not address such consequences. These concerns could limit the acceptability of employer-initiated genetic testing accessed at the workplace.

Twenty two percent of respondents had prior experience with a genomic test and 42% had accessed ancestry testing. This suggests a familiarity with genetic testing in this population. However, the preference for wGT suggests that at least some employees informed about genetic testing are not averse to genetic testing at the workplace.

In this study, most respondents were interested in wGT likely because of its perceived medical value which raises questions about why testing in the doctor’s office scenario was not the most popular choice.

It is possible that respondent’s perceptions of potential out of pocket cost considerations may have influenced expressed preferences for wGT over testing in a doctor’s office. Further, respondents indicated that privacy and confidentiality risks were least concerning for wGT. Participants may have felt that there were fewer privacy and confidentiality risks related to wGT because wGT test results are not likely to be automatically placed in a medical record. The specific basis for the preferences and assumptions about privacy and confidentiality risk are not elucidated by this survey. If validated in other settings it will be important to understand the basis of these observations. In this survey, the difference between wGT and a doctor’s office in terms of test interest was not trivial: 70 vs. 54%, with just 20% preferring DTC genetic testing. This level of interest in wGT may be unique to this study population and could be attributed to a possible familiarity of JAX employees with genomic testing, given the genetics research focus of the institution. It might also reflect unusually high levels of trust in their employer, or a belief that JAX would be particularly well suited to offer wGT. The role of employees’ trust in their employer in pursuing or declining wGT needs to be further evaluated as it is reported that a higher level of trust is associated with higher uptake of genetic testing (Boddington, 2009; Roberts et al., 2018). However, trust in the employer may not be the only factor since counterintuitively, respondents employed at JAX for over 10 years seemed less interested in wGT.

There was an apparent inverse relationship between educational status and preference for wGT among individuals who completed this survey. It is possible that those with lower levels of education might have perceived a higher potential for benefit from wGT, given that they might lack access to genetic testing or might face higher out-of-pocket costs compared with those with higher levels of education. Conversely, those with lower educational status may not have fully explored the risks of wGT. The basis for this inverse relationship and its implications regarding implementation of wGT are not elucidated in this study but constitute a potentially important avenue of exploration in future studies.

Respondents generally reported higher agreement with statements about perceived test benefits than with statements about perceived risks and concerns, although a subset of respondents expressed concerns regarding privacy and confidentiality and data use by outside groups. These findings are in accord with the outcome of a previously published employee survey that showed many employees are inclined to pursue wGT provided their genetic data would be kept safe^1^. These factors included easy and affordable access to the test with strict privacy and data ownership, allowing test result access only to the employee and their doctor. Some respondents were unsure about their appraisal of perceived benefits and risks or concerns regarding genomic testing in the three hypothetical scenarios. Taken together, these findings suggest a role for employee education in wellness programs considering incorporation of wGT, to inform employees about the long-term risks associated with wGT and to provide opportunities for the employees to assess the potential for protections to mitigate this risk.

High levels of perceived benefit or low levels of perceived risk were associated with an increased interest in wGT. However, it remains to be seen whether these findings can predict actual future utilization of wGT, as a sizeable number of respondents (20%) indicated that there were factors that would make them more comfortable or confident about pursuing wGT. These factors included guarantees about privacy and confidentiality, and assurances that test results would not be shared, and health insurance costs would not be increased.

Prior studies on prostate cancer (Harris et al., 2009) and colorectal cancer (Kinney et al., 2000) have shown an inverse association between age and interest in genetic testing. This study also demonstrated an inverse relationship between age and interest in wGT. These observations may be due to a general tendency for younger individuals to be comfortable with adopting new technology because of its increased visibility in current culture and healthcare (Khan and Mittelman, 2018). Older individuals may also prefer or be unable to prioritize genetic testing due to a variety of competing health and retirement related issues (Harding et al., 2017). Also, screening of older individuals might or might not yield the same perceived or actual benefits as wGT of younger individuals (Waltz et al., 2018). Alternatively, it may reflect a lack of understanding of existing genetic privacy and discrimination concerns related to genomic testing in workplace settings (Hudson and Pollitz, 2017; Clayton et al., 2018; Golinghorst and Prince, 2020).

There is little published data regarding employees’ preferences for return of wGT results, including the potential role of genetic counselors in wGT (Hall and Rich, 2000; Brandt-Rauf et al., 2011; McDonald et al., 2020). The results of this study suggest that a majority (55%) of those interested in wGT would like to receive their results in a meeting with a genetic counselor. A prior study on ELSI of wGT emphasized the inclusion of genetic counseling as a minimum requirement for implementing wGT (Brandt-Rauf and Brandt-Rauf, 2004). Thus, our findings reinforce this recommendation as a preference in an employee cohort.

### Limitations and Future Directions

This study has several limitations. First, the study population represents a convenience sample of employees from a single, U.S. based biomedical research institution whose mission is focused on genetics and genomics. Also, the study sample was predominantly white (88%) and therefore does not reflect the diversity of the general U.S. population. The generalizability of these findings to other settings or populations may be limited, risking over-interpretation of the results and, taken together, emphasize the need for a larger study with a more socio-demographically diverse, randomly selected sample in different workplace settings especially those settings where the mission of the workplace is not in biomedical research. Second, the study used hypothetical scenarios to simulate real-world situations in which genetic testing for disease risk might be offered. Historically wGT has been thought of in the context of testing as a condition of employment—which is inherently discriminatory (Draper, 1999). In the current wGT mode, the employer offers such testing as a part of a benefits package, often in the context of workplace wellness programs, rather than as a precondition for employment. In the context of wellness programs, employees do not need to pay for the test. This may have influenced some respondents’ choice of testing scenarios. Additionally, unless actively shared with their medical provider, wellness program-based wGT results are not included in the employees’ medical records (see Supplementary Table S5). Third, the response rate to the survey was modest, raising the possibility of non-response bias affecting the representativeness of participants’ views on wGT. Assessing non-responders in a meaningful way was beyond the scope of this research survey as the IRB would not have permitted such assessment. Fourth, the study did not evaluate the perceived or real trust of employees in institutional leadership, which might influence employees’ interest in pursuing or declining wGT. Fifth, demographic factors such as marital status or dependents and campus location across different states were not analyzed in the study. While there are limitations of this study, to the author’s knowledge these are the first data of this kind available for hypothesis generation for future experimental testing.

## Conclusion

This study of wGT provides evidence that health-related genetic testing in a workplace setting is likely acceptable by the workforce, and that many employees may prefer wGT over receiving testing from a physician or a DTC company. Additionally, the study provides evidence suggesting that younger employees may be more interested in wGT, and that lower educational status does not adversely affect interest in wGT. This study should be viewed as hypothesis generating for future studies that explore ELSI implications of wGT in other workplace settings. For example, this work should be replicated in multiple types of participant populations, perhaps using different sampling frames for ascertaining participants other than through the workforce. As the uptake of wGT continues to increase, additional studies using mixed methods approaches in different employment and demographic settings will be needed to validate the observations in this study in a real-world context to assess the actual demand for wGT and to evaluate whether any perceived or real interest in wGT can translate into better health outcomes in diverse workforces. Such studies will also need to assess: (1) the workplace culture for any potential coercive conditions; and (2) diversity of both employees and organization’s leadership on how this affects the dynamics of wGT; (3) participants’ trust in their employer; and (4) the factors that influence executive leadership’s decisions regarding offering wGT to employees.

## Data Availability Statement

The raw data supporting the conclusions of this article will be made available by the authors, without undue reservation.

## Ethics Statement

The studies involving human participants were reviewed and approved by The Jackson Laboratory Institutional Review Board. Written informed consent for participation was not required for this study in accordance with the national legislation and the institutional requirements.

## Author Contributions

KS, EL, JSR, and CL conceived the idea for the manuscript. KS drafted the manuscript and led the analyses, which was also completed by WF, DM, AP, LLP, and KB. All authors revised drafts, approved the final version, and agreed to be accountable for all aspects of the work.

## Conflict of Interest

The authors declare that the research was conducted in the absence of any commercial or financial relationships that could be construed as a potential conflict of interest.

## Footnotes

1 https://www.prnewswire.com/news-releases/65-of-employees-would-pay-for-genetic-testing-from-their-employer-sponsored-health-account-wamberg-genomic-survey-300560945.html

2 https://www.kff.org/other/state-indicator/total-population/?dataView=1&currentTimeframe=0&sortModel=%7B%22colId%22:%22Location%22,%22sort%22:%22asc%22%7D

3 https://www.kff.org/report-section/ehbs-2019-summary-of-findings/#fn

## References

[B1] Annas G. J. (2001). The limits of state laws to protect genetic information. *N. Engl. J. Med.* 345 385–388. 10.1056/nejm200108023450523 11484711

[B2] Boddington P. (2009). The ethics and regulation of direct-to-consumer genetic testing. *Genome Med.* 1:71. 10.1186/gm71 19638186 PMC2717397

[B3] Brandt-Rauf P. W. Brandt-Rauf S. I. (2004). Genetic testing in the workplace: ethical, legal, and social implications. *Annu. Rev. Public Health* 25 139–153. 10.1146/annurev.publhealth.25.101802.123012 15015916

[B4] Brandt-Rauf S. I. Brandt-Rauf E. Gershon R. Brandt-Rauf P. W. (2011). The differing perspectives of workers and occupational medicine physicians on the ethical, legal and social issues of genetic testing in the workplace. *New Solut.* 21 89–102. 10.2190/ns.21.1.j 21411427

[B5] Burke W. Trinidad S. B. Schenck D. (2019). Can precision medicine reduce the burden of diabetes? *Ethn. Dis.* 29(Suppl. 3), 669–674. 10.18865/ed.29.s3.669 31889772 PMC6919975

[B6] Clayton E. W. Halverson C. M. Sathe N. A. Malin B. A. (2018). A systematic literature review of individuals’ perspectives on privacy and genetic information in the United States. *PLoS One* 13:e0204417. 10.1371/journal.pone.0204417 30379944 PMC6209148

[B7] Dabney B. J. (1981). The role of human genetic monitoring in the workplace. *J. Occup. Med.* 23 626–631. 10.1097/00043764-198109000-00011 7024486

[B8] Drabiak K. (2017). Caveat emptor: how the intersection of big data and consumer genomics exponentially increases information privacy risks. *Health Matrix* 27:143.

[B9] Draper E. (1999). The screening of america: the social and legal framework of employers’ use of genetic information. *Berkeley J. Employ. Labor Law* 20, 286–324.

[B10] Golinghorst D. R. Prince A. (2020). A survey of U. S. state insurance commissioners concerning genetic testing and life insurance: Redux at 27. *J. Genet. Couns.* 29 928–935. 10.1002/jgc4.1197 31850620 PMC7299795

[B11] Green R. C. Lautenbach D. McGuire A. L. (2015). GINA, genetic discrimination, and genomic medicine. *N. Engl. J. Med.* 372 397–399. 10.1056/nejmp1404776 25629736

[B12] Hall M. A. Rich S. S. (2000). Genetic privacy laws and patients’ fear of discrimination by health insurers: the view from genetic counselors. *Law Med. Ethics* 28 245–257. 10.1111/j.1748-720x.2000.tb00668.x 11210377

[B13] Harding K. Mersha T. B. Webb F. A. Vassalotti J. A. Nicholas S. B. (2017). Current state and future trends to optimize the care of african americans with end-stage renal disease. *Am. J. Nephrol.* 46 156–164. 10.1159/000479479 28787724 PMC5827929

[B14] Harris J. N. Bowen D. J. Kuniyuki A. McIntosh L. FitzGerald L. M. Ostrander E. A. (2009). Interest in genetic testing among affected men from hereditary prostate cancer families and their unaffected male relatives. *Genet Med.* 11 344–355. 10.1097/gim.0b013e31819b2425 19346959 PMC2683189

[B15] Hollands G. J. French D. P. Griffin S. J. Prevost A. T. Sutton S. King S. (2016). The impact of communicating genetic risks of disease on risk-reducing health behaviour: systematic review with meta-analysis. *BMJ* 352:i1102. 10.1136/bmj.i1102 26979548 PMC4793156

[B16] Hudson K. L. Pollitz K. (2017). Undermining genetic privacy? employee wellness programs and the law. *N. Engl. J. Med.* 377 1–3. 10.1056/nejmp1705283 28537794

[B17] Khan R. Mittelman D. (2018). Consumer genomics will change your life, whether you get tested or not. *Genome Biol.* 19:120. 10.1186/s13059-018-1506-1 PMC6100720 30124172

[B18] Kinney A. Y. Choi Y. A. DeVellis B. Millikan R. Kobetz E. Sandler R. S. (2000). Attitudes toward genetic testing in patients with colorectal cancer. *Cancer Pract.* 8 178–186. 10.1046/j.1523-5394.2000.84008.x 11898257

[B19] Lappé M. (1983). Ethical issues in testing for differential sensitivity to occupational hazards. *J. Occup. Med.* 25 797–808. 6644390

[B20] Lerman C. Narod S. Schulman K. Hughes C. Gomez-Caminero A. Bonney G. (1996). BRCA1 testing in families with hereditary breast-ovarian cancer. A prospective study of patient decision making and outcomes. *JAMA* 275 1885–1892. 10.1001/jama.275.24.1885 8648868

[B21] Lieberman C. (2019). *What Wellness Programs Don’t do for Workers. Harvard Business Review.* Available online at: https://hbr.org/2019/08/what-wellness-programs-dont-do-for-workers [accessed December 10, 2020].

[B22] MacDonald C. Williams-Jones B. (2002). Ethics and genetics: susceptibility testing in the workplace. *J. Bus. Ethics* 35 235–241. 10.1002/9781119183020.ch17 12408127

[B23] McDonald W. S. Wagner J. K. Deverka P. A. Woods L. A. Peterson J. F. Williams M. S. (2020). Genetic testing and employer-sponsored wellness programs: an overview of current vendors, products, and practices. *Mol. Genet. Genomic Med.* 8:e1414. 10.1002/mgg3.1414 PMC7549551 32715662

[B24] Roberts M. C. Taber J. M. Klein W. M. (2018). Engagement with genetic information and uptake of genetic testing: the role of trust and personal cancer history. *J. Cancer Educ.* 33 893–900. 10.1007/s13187-016-1160-9 28105554

[B25] Singer N. (2018). *Employees Jump at Genetic Testing. is That a Good Thing?” The New York Times.* Available online at: https://www.nytimes.com/2018/04/15/technology/genetic-testing-employee-benefit.html (accessed at December 10, 2020).

[B26] Song Z. Baicker K. (2019). Effect of a workplace wellness program on employee health and economic outcomes: a randomized clinical trial. *JAMA* 321 1491–1501. 10.1001/jama.2019.3307 30990549 PMC6484807

[B27] Van Damme K. Casteleyn L. (1998). Ethical, social and scientific problems related to the application of genetic screening and genetic monitoring for workers in the context of a European approach to health and safety at work. *Med. Lav.* 89(Suppl. 1), S3–S72. 9673108

[B28] Waltz M. Cadigan R. J. Prince A. Skinner D. Henderson G. E. (2018). Age and perceived risks and benefits of preventive genomic screening. *Genet Med.* 20 1038–1044. 10.1038/gim.2017.206 29215654 PMC5991986

[B29] Yanes T. Willis A. M. Meiser B. Tucker K. M. Best M. (2019). Psychosocial and behavioral outcomes of genomic testing in cancer: a systematic review. *Eur. J. Hum. Genet.* 27 28–35. 10.1038/s41431-018-0257-5 30206354 PMC6303287

