# Supplementary material for: Employees’ Views and Ethical, Legal, and Social Implications Assessment of Voluntary Workplace Genomic Testing
Source: Front Genet. 2021 Mar 17;12:643304. doi: 10.3389/fgene.2021.643304 (PMC8010177; doi:10.3389/fgene.2021.643304)
Supplement: Supplementary file 1 [file Data_Sheet_1.docx]

**Working Title:**

Employees’ perspectives on taking an employer-sponsored genomic screening test

**QUESTIONS**

1. **Have you ever taken any genomic test** (a lab test that looks at lot of genes)**?**

- No
- Yes
- Unsure
- Prefer not to answer

*Add Display logic in Qualtrics – If “Yes” to Q1*

1. **If yes, what was the primary reason to take this genomic test?**

- Diagnostic – detect if there is a disease
- Predictive – detect if there is an increased risk of a disease
- Carrier testing – detect if there is a gene change linked to a disease
- Prenatal testing – detect if child has a disease prior to being born
- Ancestry – provide insights on family history and origins
- Lifestyle – provide recommendations on diet and exercise
- Unsure
- Other (Please specify): _____________

*Add Display logic in Qualtrics – If “No” to Q1*

1. **Have any of your family members ever taken any genomic test** (a lab test that looks at lot of genes)**?**

- No
- Yes
- Unsure
- Prefer not to answer

*Add Display logic in Qualtrics – If “Yes” to Q3*

1. **If yes, what was the primary reason to take this genomic test?**

- Diagnostic – detect if there is a disease
- Predictive – detect if there is an increased risk of a disease
- Carrier testing – detect if there is a gene change linked to a disease
- Prenatal testing – detect if child has a disease prior to being born
- Ancestry – provide insights on family history and origins
- Lifestyle – provide recommendations on diet and exercise
- Unsure
- Other (Please specify): _____________

______ *Page Break ______________*

In this survey, the following three imaginary scenarios will be presented to you with a common set of questions:

1. Doctor’s office-offered genomic screening test
2. Employer-sponsored genomic screening test
3. Commercially available genomic screening test

*(color formatting may appear different because of different color options in MS WORD and Qualtrics)*

**Keep in mind: You DO NOT have to take any genomic test and none will be offered to you to complete this survey.**

**Your responses will not be linked to you or affect your current or future employment at JAX.**

In this survey**,** a **“genomic screening test”** means testing your DNA from a **saliva sample** to look for **changes or mutations in a number of genes** that are known to **increase risk** for developing various diseases such as **some cancers, heart conditions, and metabolic disorders**. Metabolic disorders have a gene change that causes an enzyme deficiency.

The genomic screening test is the same in all three scenarios. How the test is being offered is different in these imaginary scenarios.

This test:

- will be done by a for-profit testing company that is NOT connected to your employer.
- will provide information on the genes and relevant conditions for which there is possible treatment or screening available.
- will not provide ancestry information.

**Imaginary SCENARIO 1: Doctor’s office-offered genomic screening test**

During a routine check-up visit, your doctor tells you about a genomic screening test offered as part of their preventive care services.

- This test looks for changes in genes related to some cancers, heart conditions, and metabolic disorders.
- There is no cost to you to get this test
- A genetic counselor at the testing company will discuss your test results with you
- The test results will be provided to you and will also go into your medical record
- The medical insurance company may have access to your test result
- Your employer will NOT know that you had this test and will not see your test results

Considering this scenario, please answer the following questions:

1. **Would you take this doctor’s office-sponsored genomic screening test?**

- Definitely not
- Probably not
- Might or might not
- Probably yes
- Definitely yes

1. Please select the level of agreement with each of the following statements. The **benefits** of a genomic screening test offered in **your doctor’s office** are….

|  | Strongly disagree | Somewhat Disagree | Neither disagree or agree | Somewhat Agree | Strongly Agree | Unsure |
| --- | --- | --- | --- | --- | --- | --- |
| Potential early disease diagnosis or detection of an increased risk for developing a disease in future |  |  |  |  |  |  |
| Timely medical intervention, if needed |  |  |  |  |  |  |
| Motivation to adopt lifestyle changes for desired health outcomes |  |  |  |  |  |  |
| Potential of getting information about the risk of health conditions for my current children or future children. |  |  |  |  |  |  |

1. Please select the level of agreement with each of the following statements. The **risks** and **concerns** related to a genomic screening test offered by **your doctor’s office** are….

|  | Strongly disagree | Somewhat Disagree | Neither disagree or agree | Somewhat Agree | Strongly Agree | Unsure |
| --- | --- | --- | --- | --- | --- | --- |
| Privacy and confidentiality of my test results |  |  |  |  |  |  |
| Use of data for any other reason or by any other person or group |  |  |  |  |  |  |
| Understanding what the test would mean to me or my family |  |  |  |  |  |  |
| Absence from work for any potential subsequent medical care |  |  |  |  |  |  |

______ *Page Break ______________*

**Imaginary SCENARIO 2: Employer-sponsored genomic screening test**

Your employer offers you an OPTIONAL genomic screening test as part of an OPTIONAL employee wellness program benefit.

- This test looks for changes in genes related to some cancers, heart conditions, and metabolic disorders.
- There is no cost to you to get this test
- A genetic counselor at the testing company will discuss your test results with you
- The test results will be provided directly to you and will NOT go into your medical record unless your share it with your doctor
- The medical insurance company will NOT have access to the test results unless you choose to put it into your medical record
- The employer will only have access to a summary of information from all participating employees and will NOT be given results of any individual employees.

**Keep in mind: You DO NOT have to take any genomic test and none will be offered to you to complete this survey. Your responses will not be linked to you or affect your current or future employment at JAX.**

Considering this scenario, please answer the following questions:

1. **Would you take this employer-sponsored genomic screening test?**
   - Definitely not
   - Probably not
   - Might or might not
   - Probably yes
   - Definitely yes
2. Please select the level of agreement with each of the following statements. The **benefits** of an **employer-sponsored genomic screening test** are….

|  | Strongly disagree | Somewhat Disagree | Neither disagree or agree | Somewhat Agree | Strongly Agree | Unsure |
| --- | --- | --- | --- | --- | --- | --- |
| Potential early disease diagnosis or identification of an increased risk for developing a disease in future |  |  |  |  |  |  |
| Timely medical intervention, if needed |  |  |  |  |  |  |
| Motivation to adopt lifestyle changes for desired health outcomes |  |  |  |  |  |  |
| Potential of getting information about the risk of health conditions for my current children or future children. |  |  |  |  |  |  |

1. Please select the level of agreement with each of the following statements. The **risks** and **concerns** related to an **employer-sponsored genomic screening test** are….

|  | Strongly disagree | Somewhat Disagree | Neither disagree or agree | Somewhat Agree | Strongly Agree | Unsure |
| --- | --- | --- | --- | --- | --- | --- |
| Privacy and confidentiality of my test results |  |  |  |  |  |  |
| Use of data for any other reason or by any other person or group |  |  |  |  |  |  |
| Understanding what the test would mean to me or my family |  |  |  |  |  |  |
| Absence from work for any potential subsequent medical care |  |  |  |  |  |  |

*Add display logic for “probably yes” or “definitely yes” or “might or might not” to Q8*

1. **Which of the following would you want to know more about before getting an employer-sponsored genomic screening test?** Check all that apply

- What my test results might mean?
- How the privacy and confidentiality of my test results will be protected?
- How the results might impact my family?
- What relevant laws and policies protect me?

*Add display logic for “probably yes” or “definitely yes” or “might or might not” to Q8*

1. **Considering results of an employer-sponsored genomic screening test, please select your response to each of the following statements.**

|  | Strongly disagree | Somewhat Disagree | Neither disagree or agree | Somewhat Agree | Strongly Agree | Unsure |
| --- | --- | --- | --- | --- | --- | --- |
| I would like to discuss my medical and family history with a genetic counselor (not accessible to employer) BEFORE pursuing this test |  |  |  |  |  |  |
| I would like to receive a copy of my test results |  |  |  |  |  |  |
| I would like to discuss my test results with a genetic counselor |  |  |  |  |  |  |
| I would like to get a letter from a genetic counselor to know the impact of my test results on my health and potentially my family's health |  |  |  |  |  |  |

*Add display logic for “probably yes” or “definitely yes” or “might or might not” to Q8*

1. **How would you prefer to receive your employer-sponsored genomic screening test result? Please rank in order of preference. (1= First choice and 6 = Last choice)**

|  | 1 | 2 | 3 | 4 | 5 | 6 |
| --- | --- | --- | --- | --- | --- | --- |
| Telephone encounter with a genetic counselor |  |  |  |  |  |  |
| Web-based encounter with a genetic counselor |  |  |  |  |  |  |
| In-person discussion with a genetic counselor |  |  |  |  |  |  |
| Email with a link to a secure web portal with test results |  |  |  |  |  |  |
| Only written summary of test result |  |  |  |  |  |  |
| Other method (please specify): |  |  |  |  |  |  |

______ *Page Break ______________*

**Imaginary SCENARIO 3: Commercially available genomic screening test**

You see a commercial from a genomic testing company that offers a home-based genomic screening test.

- This test looks for changes in genes related to some cancers, heart conditions, and metabolic disorders.
- This commercially available test will cost you about $200 - $400
- A genetic counselor at the testing company is available to discuss your test results with you
- The test results will NOT go into your medical record unless your share it with your doctor
- The medical insurance company will NOT have access to the test results unless you choose to put it into your medical record
- Your employer will NOT know that you had this test unless you specify

Considering this scenario, please answer the following questions:

1. **Would you take this commercially available genomic screening test?**
   - Definitely not
   - Probably not
   - Might or might not
   - Probably yes
   - Definitely yes
2. Please select the level of agreement with each of the following statements. The **benefits** of a **commercially available genomic screening test** are….

|  | Strongly disagree | Somewhat Disagree | Neither disagree or agree | Somewhat Agree | Strongly Agree | Unsure |
| --- | --- | --- | --- | --- | --- | --- |
| Potential early disease diagnosis or identification of an increased risk for developing a disease in future |  |  |  |  |  |  |
| Timely medical intervention, if needed |  |  |  |  |  |  |
| Motivation to adopt lifestyle changes for desired health outcomes |  |  |  |  |  |  |
| Potential of getting information about the risk of health conditions for my current children or future children. |  |  |  |  |  |  |

1. Please select the level of agreement with each of the following statements. The **risks** and **concerns** related to a **commercially available genomic screening test** are….

|  | Strongly disagree | Somewhat Disagree | Neither disagree or agree | Somewhat Agree | Strongly Agree | Unsure |
| --- | --- | --- | --- | --- | --- | --- |
| Privacy and confidentiality of my test results |  |  |  |  |  |  |
| Use of data for any other reason or by any other person or group |  |  |  |  |  |  |
| Understanding what the test would mean to me or my family |  |  |  |  |  |  |
| Absence from work for any potential subsequent medical care |  |  |  |  |  |  |

______ *Page Break ______________*

1. **Based on the three imaginary scenarios, which scenario would you be most comfortable with to take a genomic screening test?**
   - Doctor’s office-offered genomic screening test – SCENARIO 1
   - Employer-sponsored genomic screening test – SCENARIO 2
   - Commercially available genomic screening test – SCENARIO 3
   - I am equally comfortable with all three scenarios
   - I am equally uncomfortable with all three scenarios
   - Unsure
   - Prefer not to answer
2. Are there any other factors that would make you **more comfortable or confident** about having an **employer-sponsored genomic screening test** as part of an optional employee wellness program benefit?
   - No
   - Maybe (please indicate what they might be) __________
   - Yes (Please indicate what are they) ________________
   - Unsure
   - Prefer not to answer

______ *Page Break ______________*

Your responses to the open-ended question will not be linked to you or your employment.

Please consider sharing your thoughts on why employer-sponsored genomic screening test is a good or bad idea.

1. **What thoughts would you like to share about the idea of an employer-sponsored genomic screening test as part of an optional employee wellness program benefit?** You may add your thoughts on **additional benefits** or **concerns** or **any other aspects** not covered in the previous questions.

_______________________________________________________________________________________________________________________________________________________________________________________________________________________________________________________________

______ *Page Break ______________*

**Demographic questions:**

- Number of year(s) employed at The Jackson Laboratory:
  - Less than a year
  - 1-2
  - 3-5
  - 6-9
  - 10-19
  - More than 20 years
- Sex:
  - Female
  - Male
  - Prefer not to answer
- Age:
  - 18-25
  - 26-35
  - 36-45
  - 46-55
  - 56 and over
- Highest level of education:
  - High school graduate
  - GED or equivalent
  - Some college, no degree
  - Associate degree: occupational, technical, or vocational program
  - Associate degree: academic program
  - Bachelor's degree (example: BA, AB, BS, BBA)
  - Master's degree (example: MA, MS, MEng, MEd, MBA)
  - Professional school degree (example: MD, DDS, DVM, JD)
  - Doctoral degree (example: PhD, EdD)
- How would you describe yourself? Please choose from the following groups:
  - American Indian or Alaska Native
  - Asian
  - Black or African American
  - Native Hawaiian or other Pacific Islander
  - White
  - More than one race
  - Prefer not to answer
- Are you Hispanic or Latino?
  - No
  - Yes
  - Prefer not to answer

We appreciate your feedback. Please click the **“submit**” button.

**SUBMIT**

*After clicking on “Submit”, participants will be taken to a separate page (separate survey in Qualtrics) to read the following text.*

Thank you for your time to complete this survey.

You are welcome to voluntarily contact __________ to share any additional thoughts.

**Your responses WILL NOT be linked to you or affect your current or future employment at JAX.**

**Useful educational links:**

Frequently Asked Questions about Genetic Testing:

<https://www.genome.gov/19516567/faq-about-genetic-testing/>

Video on difference between genetics vs. genomics:

<https://www.jax.org/personalized-medicine/precision-medicine-and-you/genetics-vs-genomics>

Video on genetic counseling by the National Society of Genetic Counselors (NSGC)

<https://www.youtube.com/watch?v=GDjLazXGV0s>
