# Supplementary material for: Employees’ Views and Ethical, Legal, and Social Implications Assessment of Voluntary Workplace Genomic Testing
Source: Front Genet. 2021 Mar 17;12:643304. doi: 10.3389/fgene.2021.643304 (PMC8010177; doi:10.3389/fgene.2021.643304)
Supplement: Supplementary file 3 [file Data_Sheet_3.docx]

Employees’ Views and Ethical, Legal, and Social Implications Assessment of Voluntary Workplace Genomic Testing

Supplementary Table 5: Imaginary scenarios covered in the survey

| **Imaginary SCENARIO 1: Doctor’s office-offered genomic screening test** | **Imaginary SCENARIO 2: Employer-sponsored genomic screening test** | **Imaginary SCENARIO 3: Commercially available genomic screening test** |
| --- | --- | --- |
| During a routine check-up visit, your doctor tells you about a genomic screening test offered as part of their preventive care services.   - This test looks for changes in genes related to some cancers, heart conditions, and metabolic disorders. - There is no cost to you to get this test - A genetic counselor at the testing company will discuss your test results with you - The test results will be provided to you and will also go into your medical record - The medical insurance company may have access to your test result - Your employer will NOT know that you had this test and will not see your test results | Your employer offers you an OPTIONAL genomic screening test as part of an OPTIONAL employee wellness program benefit.   - This test looks for changes in genes related to some cancers, heart conditions, and metabolic disorders. - There is no cost to you to get this test - A genetic counselor at the testing company will discuss your test results with you - The test results will be provided directly to you and will NOT go into your medical record unless your share it with your doctor - The medical insurance company will NOT have access to the test results unless you choose to put it into your medical record - The employer will only have access to a summary of information from all participating employees and will NOT be given results of any individual employees.   **Keep in mind: You DO NOT have to take any genomic test and none will be offered to you to complete this survey. Your responses will not be linked to you or affect your current or future employment at JAX.** | You see a commercial from a genomic testing company that offers a home-based genomic screening test.   - This test looks for changes in genes related to some cancers, heart conditions, and metabolic disorders. - This commercially available test will cost you about $200 - $400 - A genetic counselor at the testing company is available to discuss your test results with you - The test results will NOT go into your medical record unless your share it with your doctor - The medical insurance company will NOT have access to the test results unless you choose to put it into your medical record   Your employer will NOT know that you had this test unless you specify |
